# The barriers and facilitators to health-promoting lifestyle behaviors among people with multiple sclerosis during the coronavirus disease 2019 pandemic: a content analysis study

**DOI:** 10.1186/s12883-022-03019-z

**Published:** 2022-12-19

**Authors:** Soheileddin Salmani, Seyyed Hossein Mousavi, Samira Navardi, Fatemeh Hosseinzadeh, Shahzad Pashaeypoor

**Affiliations:** 1 Department of Nursing, Rozbeh Hospital, Tehran University of Medical Sciences, Tehran, Iran; 2 School of Nursing and Midwifery, Tehran University of Medical Sciences, Tehran, Iran; 3 Multiple Sclerosis Research Center, Neuroscience Institute, Tehran University of Medical Sciences, Tehran, Iran; 4 Department of Community Health Nursing, School of Nursing and Midwifery, Tehran University of Medical Sciences, Tehran, Iran; 5 Department of Community Health and Geriatric Nursing, School of Nursing and Midwifery, Tehran University of Medical Sciences, Tehran, Iran; 6 Community Based Participatory Research Center, Iranian Institute for Reduction of High – Risk Behaviors,, Tehran University of Medical Sciences, Tehran, Iran

**Keywords:** Multiple sclerosis, Health-promoting lifestyle, Barriers, Facilitators

## Abstract

**Background and aim:**

Health-promoting lifestyle behaviors (HPLBs) have a significant impact on disease management among people with multiple sclerosis (MS). However, the coronavirus disease 2019 (COVID-19) pandemic has significantly affected lifestyle of all individuals, particularly patients with chronic diseases. The present study aimed to explore the barriers and facilitators to HPLBs among people with MS during the COVID-19 pandemic.

**Methods:**

This qualitative study was conducted in Iran. Participants were sixteen people with MS purposively selected from the central MS clinic of a referral specialty neuroscience hospital in Tehran, Iran. Data were collected via in-depth semi-structured face-to-face interviews and concurrently analyzed through conventional content analysis.

Findings: The mean of participants’ age was 37.93 years and most participants were female (81.25%). The major barriers to HPLBs were lack of knowledge, limited access to resources, and poor health status, while the major facilitators were attention to inner abilities and social support.

**Conclusion:**

Many different factors such as lack of knowledge, limited access to resources, poor health status, awareness, and social support can influence engagement in HPLBs among people with MS. Healthcare authorities and policymakers need to use quality educational and supportive interventions to improve knowledge, health literacy, perceived support, self-efficacy, and self-care ability among people with MS during the COVID-19 pandemic.

**Supplementary Information:**

The online version contains supplementary material available at 10.1186/s12883-022-03019-z.

## Introduction

Multiple sclerosis is a significant global health challenge of the modern world [1]. It is a prevalent chronic and progressive disorder of the central nervous system among young adults [2]. Its underlying cause is still unknown, but its potential risk factors are genetic background, immune deficiency, environmental factors, viral infections, vitamin D deficiency, smoking, and psychological strains [2]. The prevalence of MS is increasing worldwide and is more than 100 in 100,000 people in North America, West Europe, and Australia, less than 30 in 100,000 people in Equatorial countries, and 148.06 in 100,000 people in Iran [3]. MS affects women three times more than men [4].

MS has an unpredictable course in most cases [5] and is associated with significant losses, changes, and problems that negatively affect the cognitive, emotional, and functional status [6]. People living with MS may lose their functioning, experience changes in their roles and abilities, and suffer from different symptoms and problems such as sensory disorders, muscle cramps, visual impairment, diplopia, fatigue, tremor, elimination problems, sexual dysfunction, numbness, and speech problems [4, 7]. MS also significantly changes data processing abilities, attention, memory, and mood [8]. The prevalence rates of psychological and neurological changes, depression, and anxiety are 40–65% [9], 30–40%, and 36% in MS [10], respectively.

Health-promoting lifestyle behaviors (HPLBs) have a significant impact on MS management [6, 11]. By definition, HPLBs are a set of behaviors that help maintain and promote health and self-actualization and form a healthy lifestyle [12]. HPLBs include proper nutrition, regular physical activity, stress management, spiritual growth, health responsibility, and interpersonal relationships. These behaviors promote the health status and reduce healthcare costs [13]. Regular physical activity, proper nutrition, treatment adherence, and stress management are extremely important for the management of chronic diseases and improvement of general well-being [14]. A study found that lifestyle modification and HPLBs helped to manage the complications of chronic diseases such as MS [11]. Evidence shows that HPLBs such as stress management, physical activity, social activities, and healthy eating can improve the cognitive, emotional, and functional status of the people living with MS, reduce their problems, and improve their quality of life [6]. Moreover, severe depression, pain, fatigue, and cognitive problems are less prevalent among individuals with healthy lifestyle [15]. These behaviors are modifiable; hence, they are considered as a key factor in effective MS management [15, 16].

Evidence suggests that adequate intake of fruits, vegetables, and grains and limited intake of sugar and red meat are associated with lower levels of disability in people with MS [15]. Regular physical activity also reduces their fatigue, depression, cognitive problems, and sensory disorders and improves balance, cardiovascular and neuromuscular function, and quality of life [17]. Effective stress management also facilitates MS management, prevents its aggravation, and reduces its symptoms [18, 19]. Moreover, social activities and interpersonal relationships are effective in reducing stress, anxiety, and depression [19].

Many different factors can affect engagement in HPLBs [20]. Epidemics, such as the current coronavirus disease 2019 (COVID-19) pandemic, are among these factors [21]. COVID-19 is a viral infectious disease caused by the SARSCoV-2 virus. The World Health Organization introduced COVID-19 as a pandemic in March 11, 2020 [22]. Its outbreak was unpredictable and was associated with significant life losses [23]. COVID-19 has affected 420 million people and caused more than 5 million deaths across the world so far [24, 25]. Strategies such as complete or partial quarantine, lockdown, and physical distancing are recommended for COVID-19 control [23]. These strategies have reduced social relationships, access to healthcare providers and services, and social support in people living with chronic diseases and have caused them varying levels of stress [18, 19] and depression [19]. Moreover, the COVID-19 pandemic has significantly affected HPLBs, changed daily habits, and increased the likelihood of engagement in unhealthy behaviors such as improper nutrition and inactivity [21, 23].

Despite the importance of HPLBs in the effective management of MS, studies have shown poor adherence to these behaviors in people living with MS. A study found that compared to healthy individuals, people with MS had a lower adherence to HPLBs such as self-actualization, interpersonal relationships, stress management, and physical activity [26]. Many different factors can facilitate or reduce their engagement in HPLBs. A qualitative study of adolescents with MS showed that the barriers to engagement in physical activity were MS symptoms such as fatigue, lack of social support from peers and family members, and time limitation while its facilitators were symptom management, adequate social support from peers and family members, and adequate time [27]. Another study showed that the debilitating symptoms of MS reduced the patients’ ability to engage in HPLBs [28]. On the other hand, a study found that improving motivation, self-efficacy, goal setting, and planning for action could positively affect long-term adherence to physical activity in people with MS [29]. Similarly, a study indicated that increased self-management and self-efficacy improved healthy eating behaviors in people with MS [30]. Moreover, the results of a study revealed that age was an influential factor on HPLBs and reported that older patients learned how to cope with their problems better over time. This study also found that perceived social support was the strongest predictor of all HPLBs except physical activity [31].

Previous studies investigating the determinants of engagement in HPLBs in people with MS only addressed some aspects of these behaviors. Moreover, most of them were conducted before the COVID-19 pandemic; hence, there is limited information in this regard during the pandemic. Therefore, the present study was conducted to explore the barriers and facilitators of HPLBs in people living with MS during the COVID-19 pandemic. Barriers and facilitators are the strongest predictors of HPLBs [32] and their identification helps to develop more effective plans to modify the patients’ lifestyle and improve their engagement in HPLBs [32].

## Methods

### Design

The present study was conducted using a conventional content analysis (CCA) approach. The study therefore follows an inductive approach, with categories emerging solely from the data meaning no pre-determined theory or attitude is required. The results of the content analysis are primarily derived from the participants’ distinctive and authentic perceptions [33]. In this way, CCA is a suitable approach to explain the barriers and facilitators of HPLBs in people living with MS. The COREQ reporting guideline was used to ensure that the methods are appropriately reported (Additional file 1: Appendix 1).

### Participants and setting

This study was conducted in the central MS clinic of a referral neuroscience hospital in Tehran, Iran in 2021. The people with MS from different cities of Iran are referred to this hospital to receive MS care services. The participants were recruited directly based on the inclusion criteria. The study population consisted of people living with MS that were referred to the study setting. Sixteen people with MS were selected purposively based on the following selection criteria: age above 18 years and a definitive diagnosis of MS at least 6 months before the study. The only exclusion criterion was the presence of acute mental or psychological disorders.

### Data collection

First, the study objectives were explained to the participants and signed written informed consent was obtained directly from those who wished to participate in the study at the beginning of the interviews. An information sheet was given to the participants and their questions were answered prior to participation. Permission was also obtained to record the interviews with an audio recorder. The participants were reassured about the principles of confidentiality and anonymity, the voluntary participation in the study, and the right to withdraw from the study at any time without any consequences.

Data were collected via in-depth semi-structured face-to-face interviews held in a medical visit room in the study setting according to the precautions for the prevention of COVID-19 transmission. The interview guide included open-ended questions such as, “Which behaviors promote your health?”, “What are the benefits of HPLBs for you?”, “What barriers do you face in the family or society to engage in HPLBs?”, “What social or environmental factors help you engage in HPLBs?”, and “What factors facilitate your engagement in HPLBs?”. The process of the interviews was flexible so that the interviewer could collect in-depth data. Accordingly, besides the main interview questions, probing questions were asked to obtain a better understanding of the participants’ narratives. Examples of these questions were “What do you mean by this?”, “Why?”, and “How?”. The interview guide was developed by the authors to address the research question. At the end of the interviews, the interviewees were asked about any non-addressed points, were informed about the potential need for more interviews, and were requested to allow the interviewer to contact them in case of any possible questions about their experiences. The interviews were continued until data saturation was achieved, i.e. no new information was obtained from the data and all aspects of the data were adequately developed [34]. All interviews were conducted by one of the researchers. In the two last interviews, the interview data became repetitive and no new data were obtained. The interviews were recorded using an audio recorder and were immediately transcribed verbatim by one of the researchers manually. In order to protect the data of the recorded interviews, the interview files remained with the corresponding author in an encrypted format.

### Data analysis

Data were analyzed using the conventional content analysis (CCA) approach. Content analysis is a qualitative data analysis approach for data categorization and a research method to describe the phenomena systematically and objectively [35]. In the present study, the conventional content analysis approach proposed by Graneheim and Lundman was used for data analysis. The five steps of this approach include transcription of the whole interview immediately after holding it, reading the transcript several times to obtain a general understanding of it, determining and coding the meaning units, categorizing the primary codes into larger categories, and determining the subjects of the categories [36]. Accordingly, the transcript of each interview was read several times to obtain a general understanding of its content. Then, meaning units were identified and coded, the codes were compared with each other regarding their similarities and differences, and codes with conceptual similarities were grouped into categories. The categories were also compared with each other and grouped into larger categories based on their conceptual similarity. At the end of each interview, the recorded information was transferred to a sheet after listening to it 2–3 times in the shortest possible time. In addition, at the end of each session, the quality of the field notes was assessed. This process was the same for all interviews. To ensure the accuracy of the information, all the information transferred to the sheet was reviewed while listening to the recorded material. The process of data analysis was appraised by the members of the research team in several sessions to reach an agreement about the accuracy of data analysis. The MAXQDA software was used for data analysis.

### Trustworthiness

Different techniques were used to ensure trustworthiness. For example, the data were independently analyzed by three authors and the extracted codes were reviewed by the research team. Then, the categories obtained from the analysis were compared with each other. Any disagreement over categories was resolved by discussion. Moreover, the codes of several interviews were provided to several participants in the process of member check to assess the accuracy of data analysis. Peer review and audit were carried out on the data. A number of interviews were randomly selected and given to some researchers that were familiar with qualitative research but were not part of the study to review the data and share their comments and feedback. It is noteworthy that the researchers tried to disregard their assumptions in the process of data collection and analysis. These steps were taken to minimise the interpretation of data and ensure that findings were truly embedded within what participants were saying. Data transferability was sought through maximum variation sampling and demonstrating the participants’ characteristics, which could also help other researchers to follow the research procedure.

## Results

The mean interview time was 30 ± 15 min. The participants included 16 people with MS with a mean age of 37.93 years. Most of the participants were female (81.25%), married (62.5%), and housewives (62.5%), had Social Security Insurance, and lived in Tehran (67.75%). More than one third of them were 30–40 years (37.5%), more than one third of them were 40–50 years (37.5%), and half of them had a high school diploma (50%) (Table 1).

**Table 1:** Participants’ characteristics

Characteristics		N (%)
Age (Years)	20–30	4 (25)
	30–40	6 (37.5)
	40–50	6 (37.5)
	Total	16 (100)
Gender	Male	3 (18.75)
	Female	13 (81.25)
	Total	16 (100)
Educational level	Guidance school	1 (6.25)
	Diploma	8 (50)
	University	7 (43.75)
	Total	16 (100)
Marital status	Single	5 (31.25)
	Married	10 (62.5)
	Divorced	1 (6.25)
	Total	16 (100)
Employment status	Unemployed	1 (6.25)
	Employed	3 (18.75)
	Housewife	10 (62.5)
	Student	2 (12.5)
	Total	16 (100)
Income (US dollars)	715–950	2 (12.5)
	950–1200	2 (12.5)
	1200–1430	3 (18.75)
	1430–1670	1 (6.25)
	Not reported	8 (50)
	Total	16 (100)
Insurance type	Social Security	11 (68.75)
	National health	3 (18.75)
	Other	2 (12.5)
	Total	16 (100)
Place of residence	Tehran	11 (68.75)
	Cities near Tehran	5 (31.25)
	Total	16 (100)

A total of 853 primary codes were generated during data analysis, which were reduced to 95 final codes after comparison of their similarities. Finally, the barriers to HPLBs were grouped into 3 main categories, including lack of knowledge, limited access to resources, and poor health status. The facilitators were grouped into 2 main categories, including attention to inner abilities and social support (Table 2).

**Table 2:** The barriers and facilitators to health-promoting lifestyle behaviors among people with MS

Semantic Units	Subcategories	Categories	Themes
Lack of sufficient knowledge about healthy lifestyle, need for knowledge about prevention of disease complications, need for knowledge about new condition management	People’s lack of knowledge	Lack of knowledge	Barriers
The family's lack of understanding of the symptoms and complications of the disease, the family members' lack of satisfaction with their inability to fulfill family roles and responsibilities	Families’ lack of knowledge		
Receiving negative vibes from friends due to lack of knowledge about the disease, trying to avoiding people around	Friends and relatives’ lack of knowledge		
The need for culturalization in society, fear of stigmas and disgusting behaviors, false beliefs in society due to not having enough knowledge about the disease	Public’s lack of knowledge		
The need for financial support and insurance, the cost of food and medicine, etc., low income and insufficient livelihood	Lack of financial resources	Limited access to resources	
Lack of sports facilities suitable for people with MS, lack of adaptation to the environment, distance	Lack of social resources		
The need for specialized and supportive services, the expectation of follow-up and support from therapists, the passivity of associations and care systems	Lack of healthcare resources		
Lack of specialized educational media	Limited access to educational resources		
Failure to perform healthy behaviors, Low ability to take care of oneself	Reduced self-care ability	Poor health status	
Complications of illness, losses due to illness	Severity of complications		
Trying to perform healthy behaviors after illness, paying attention to the symptoms of illness and your health, trying to manage medical complications	Self-efficacy	Attention to inner abilities	Facilitators
More communication with God, taking refuge in spirituality, creating solitude and gratitude	Spirituality		
Dreams for the future, love for life, hope for the future	Hopefulness		
Support of family and spouse, being motivated by spouse, love in family	Family support	Social support	
Communication with friends, meetings with peers, energizing friendships	Peer support		
Using cyberspace, media as an educational resource	Informational support		
Relationships with healthcare providers, support associations	Healthcare support		
Supporting charities, creating recreational spaces for people with MS, financial support	Charity support		

### Barriers to HPLBs in people with MS

The three main barriers to HPLBs among people with MS were lack of knowledge, limited access to resources, and poor health status.

#### Lack of knowledge

MS frequently has a chronic and progressive course and is associated with many different consequences [2]. Therefore, people living with MS and their caregivers need to have adequate knowledge about MS, its management, prevention of its potential complications, and strategies for health promotion. However participants reported a lack of knowledge to be a barrier to HPLB’s as described below. This category had 4 subcategories, including people’s lack of knowledge, families’ lack of knowledge, friends and relatives’ lack of knowledge, and public’s lack of knowledge.

##### People’s lack of knowledge

Lack of knowledge about behavior management, healthy lifestyle, complications prevention, and management of new conditions was among the barriers to HPLBs. The participants stated that they had limited knowledge about HPLBs and highlighted that the COVID-19 pandemic had created new conditions for them that increased their need for up-to-data knowledge in order to effectively manage their conditions.

I am really confused about this new condition. I don’t know what to eat, whether I should have a specific diet during COVID-19, and how much this new condition will affect my health status (P. 5).

##### Families’ lack of knowledge

Family members are key individuals in providing care for people with chronic conditions such as MS [37]. Therefore, they need to have adequate knowledge about patient care and health promotion. However, the participants mentioned their family members’ poor understanding and acceptance of MS symptoms and complications, dissatisfaction with MS-related family conditions, and inability to assume family roles and responsibilities as the barriers to HPLBs. They argued that they could not perform their roles and responsibilities in the family and their family members had a poor understanding of their symptoms and complications, had problems accepting their conditions, and were dissatisfied with MS-related family conditions.

I get tired easily when I work. My spouse complains why I get tired so soon. They think that I’m healthy as long as I can walk. They have a poor understanding of the inside of me and my disease (P. 1).

##### Friends and relatives’ lack of knowledge

Friends and relatives’ negativism, colleagues’ poor understanding of MS, and attempts of people with MS to distance from others were among the barriers to HPLBs. The participants noted that their friends and relatives had negative attitudes towards MS and hence they tried to stay away from them. Moreover, the participants reported that their colleagues’ poor understanding of their conditions negatively affected their engagement in HPLBs.

I have found that others are very important for effective MS management. I mean that I should not have close relationship with negative individuals and should eliminate them from my life (P. 6).

Well, I know that many patients are not appropriately understood and therefore experience stress and humiliation (P. 7).

##### Public’s lack of knowledge

The participants stated that public’s lack of knowledge and misconceptions about MS resulted in stigmatization and unacceptable behaviors.

I think only four people know my MS. I didn’t tell anybody about it because they show bad behaviors as soon as they find out about it. The public’s limited knowledge, misunderstanding, misconceptions, and poor attitude are associated with stress and anxiety. I think people need to know more about diseases (P. 3).

#### Limited access to resources

The management of chronic diseases and promotion of the health status need multiple resources [37]. However, the COVID-19 limited the access of people with chronic conditions such as MS to healthcare resources and services. Therefore, they need to perform the majority of care measures at home. This category had 4 subcategories, including lack of financial resources, lack of social resources, lack of healthcare resources, and limited access to educational resources.

##### Lack of financial resources

Living with MS may lead to employment loss, income reduction, and financial problems and hence reduces the patients’ ability to purchase healthy foods and medications [38]. The COVID-19 pandemic has also limited their occupational activities, income, and access to resources. Therefore, the participants need MS-compatible employment, adequate financial support, and insurance coverage to be able to afford their costs.

Everything is really expensive. An item that previously cost 200,000 Tomans is now 500,000–600,000 Tomans. This limits our ability to spend on health (P. 5).

##### Lack of social resources

The participants noted that lack of MS-compatible sports facilities, poor environmental design, and long distances limited their ability to engage in HPLBs such as physical activity.

COVID-19 limited our ability to use sports facilities such as sports clubs. I wish the clinics provided us with some sports facilities. We have almost no physical activity, which is not good at all (P. 2).

##### Lack of healthcare resources

Lack of specialized healthcare and support services was another barrier to HPLBs in the study participants. The COVID-19 pandemic also limited their access to healthcare services and forced them to rely more on self-care at home.

We expected to have the opportunity to exercise in an appropriate environment during the COVID-19. However, restrictions due to COVID-19 limited us, nobody supported us, and the healthcare system did not provide us with an appropriate environment (P. 7).

##### Limited access to educational resources

Although patients with chronic diseases need continuous specialized education, the participants reported limited access to quality educational resources.

There are limited educational programs about MS in the social media. Healthcare programs needed to pay more attention to us during the COVID-19 pandemic. We really did not know where we could find answers to our questions (P. 10).

##### Poor health status

The third main barrier to HPLBs in people with MS was their poor health status. Living with MS and MS-related consequences and limitations reduces their self-care ability and ability to pay attention to other aspects of their health. This category had 2 subcategories including reduced self-care ability and severity of complications.

##### Reduced self-care ability

The participants reported low self-care ability and limited motivation for self-care and behavior modification due to low self-efficacy and lack of appropriate incentives.

Sometimes I don't have enough energy and motivation to cook or exercise. I get disappointed, experience weakness, and feel that I can’t do anything (P. 5).

##### Severity of complications

The participants stated that the complications of MS, their inability to manage MS symptoms, and different MS-related losses reduced their ability to engage in HPLBs.

Extreme fatigue has made me unable to perform my daily activities. The COVID-19 pandemic also aggravated my condition. I also lost my job. I am not in the mood for sports activities (P. 8).

### Facilitators of HPLBs in people with MS

The two main categories of the facilitators of HPLBs in people with MS were attention to inner abilities and social support.

#### Attention to inner abilities

Attention to inner abilities may be a key factor in the effective management of chronic diseases such as MS. Using inner abilities can be a way to deal with life's challenges. This category had 3 subcategories, including self-efficacy, spirituality, and hopefulness.

##### Self-efficacy

Self-efficacy helps individuals to cope with their diseases and conditions [1]. The participants who paid closer attention to their symptoms and health and tried to manage their disease complications more actively engaged in HPLBs.

Previously, I never followed any health-related recommendations because I was genetically strong, did heavy tasks, ate whatever available, did not exercise, and sometimes smoked. But now, I have MS and need to observe many things such as smoking cessation and healthy nutrition (P. 10).

##### Spirituality

The participant reported that relationship with God, giving thanks to Him, and seclusion improved their ability to engage in HPLBs.

I felt that a stronger relationship with God improved my energy and calmness. Previously, I never recited the holy Quran; but now I recite it frequently because it has been associated with positive outcomes (P. 2).

##### Hopefulness

Hopefulness about the future, love of life, and consideration of wishes improved participants’ attitudes towards HPLBs and encouraged them to engage in them.

My wishes motivate me and help me feel better (P. 6).

#### Social support

Social support, including emotional, instrumental, and informational components, is a significant factor that helps patients cope with their disease. This category had 5 subcategories, including family support, peer support, informational support, healthcare support, and charity support.

##### Family support

Support and encouragement from family members and their love facilitated participants’ engagement in HPLBs.

My spouse supports me with his calmness, behaviors, conduct, and good understanding of my conditions. He is now the only source of support for me (P. 8).

##### Peer support

The participants stated that friendly relationships with peers, significant social contribution, and participation in peer meetings facilitated their engagement in HPLBs.

Previously, they arranged tours to visit different places like exhibitions. Such programs were very helpful and provided patients with the opportunity to get acquainted and talk with each other (P. 12).

##### Informational support

The participants reported that the cyberspace and media advertisements not only helped them improve their knowledge, but also positively affected their engagement in HPLBs, particularly during the COVID-19 pandemic.

Since the outbreak of COVID-19, I’ve searched the Internet for the different types of physical exercises, such as stretching or balance exercises and performed them based on the available instructions (P. 5).

##### Healthcare support

Relationships with healthcare providers and MS support associations also facilitated engagement in HPLBs.

I really integrated sports into my life. The association holds online yoga sessions for us once a week on Mondays. It is very helpful (P. 7).

##### Charity support

Charity organizations provided participants with recreational facilities and financial support and hence facilitated their engagement in HPLBs.

I called the charity organization and told them that I needed a drug. Its name was Zytux. They asked me to send them my prescription through WhatsApp. Two days later, they sent the medication for free. I thanked them very much. Charity organizations are a big help in this COVID-19 condition (P. 3).

## Discussion

The aim of the present study was to explore the HPLBs barriers and facilitators in people with MS during the COVID-19 pandemic. Barriers had 3 main categories, including lack of knowledge, limited access to resources, and poor health status, and facilitators had 2 main categories, including Attention to inner abilities and social support.

### Barriers to HPLBs in people with MS

One of the main barriers of HPLBs among people with MS was lack of knowledge, which had 4 subcategories, including people’s lack of knowledge, families’ lack of knowledge, friends and relatives’ lack of knowledge, and public’s lack of knowledge. Although people with MS actively seek lifestyle-related information, there are limited management guidelines or strategies that include education and support for lifestyle and risk factor modification [39]. A review study reported low levels of physical activity and exercise in people with MS due to barriers such as lack of knowledge [38]. Another study showed that while adequate knowledge about physical activity could be a significant determinant of adherence to physical activity, people with MS had limited knowledge about appropriate physical activities and needed education about the positive effects of physical activity on their general symptoms, health, and social contribution [40]. Similarly, a study found lack of knowledge as a main barrier to engagement in physical activity in people with MS [41]. The results of these studies are consistent with the present research. In the current study, the people with MS mentioned that the Covid-19 pandemic created new conditions for them that required training and empowerment for HPLBs. It seems that the use of virtual training in pandemics can help improve the lifestyle in people with chronic diseases [22, 23]. Lack of knowledge is associated with negative consequences such as dissatisfaction with marital relationship [42], while continuous and adequate training for people with chronic diseases such as MS about their diseases and strategies to manage the risk factors of their disease has many positive outcomes [39]. For example, it reduces fear, anxiety, and depression and improves the quality of life of family members, strengthens marital relationships, and facilitates successful coping among patients [42].

Our findings also showed that the family members’ poor understanding of the patients’ conditions was a barrier to HPLBs. COVID-19-related conditions have led to severe stress for families and affected the relationships of family members. Promotion of stress management techniques is therefore recommended for this population and their families during the COVID-19 pandemic [19]. Our participants also reported public’s lack of knowledge, poor acceptance, and stigmatization as barriers to their HPLBs. Stigmatization negatively affects the quality of life, perceived psychosocial support, and interpersonal relationships with family members and physicians [43].

Limited access to resources was the second main barrier to HPLBs in people with MS. This category had 4 subcategories including lack of financial resources, lack of social resources, lack of healthcare resources, and limited access to educational resources. Chronic diseases can affect different aspects of family life including daily life routines and financial and occupational decisions [37]. A study reported logistical problems such as limited access to financial and supportive resources as factors contributing to limited physical activity and exercise in people with MS [38]. COVID-19-related conditions can also aggravate financial problems of people with MS; these conditions cause stress and exacerbate the disease since COVID-19 has had negative effects on many aspects of educational, occupational, and financial activities and increased unemployment rate [44]. It is consistent with the results of the present study.

Our results also showed that COVID-19-related restrictions and quarantine reduced the participants’ engagement in physical activity due to restricting their access to the available resources for physical activity. Access to physical activity resources is a predictor of long-term engagement in physical activity [40]. A study found that geographical distance was a main barrier to engagement in physical activity in people with MS [39]. Inability to access the resources for physical activity and exercise during the COVID-19 pandemic reduced patients’ access to other facilities, limited their social relationships, and decreased their social support. It also deprived them of the positive effects of physical activity on their stress management ability. The present study also showed that the patients’ inability to have regular relationships with healthcare providers, particularly during the COVID-19 pandemic, was a major barrier to their engagement in HPLBs. Healthcare providers’ support is a key factor in disease management in people with MS [40]. However, during the pandemic, most of the hospitals shifted their services towards COVID-19 care; hence, they could not provide chronically ill patients with adequate services [45]. Moreover, the risk of COVID-19 required these patients to stay at home and satisfy most of their needs through self-care. Limited access to educational resources was another factor contributing to the lack of knowledge and another barrier to HPLBs in people with MS in the present study. A study conducted in Iran showed the insignificant contribution of media to providing education about MS [46]. Two other studies also found poor public knowledge about MS and argued that mass media were a suitable route for improving public knowledge about MS [47, 48].

Poor health status was the third main category of the barriers to HPLBs in people with MS, which had two subcategories including reduced self-care ability and severity of complications. MS symptoms and complications such as mental or physical fatigue and disability are among the major barriers to the adoption of a healthy lifestyle [39]. Previous studies also showed that MS-related problems such as fatigue, reduced mobility, symptom fluctuations, fear, horror, and poor self-management were significant barriers to engagement in physical activity [38, 40, 49] and healthy eating [49].

The findings of the present study also revealed that lack of motivation for self-care was a barrier to engagement in HPLBs. Motivation can be defined as “the impetus that gives purpose or direction to behavior and operates at a conscious or unconscious level in humans” [40]. In line with our findings, a previous study reported lack of motivation as a major barrier to the adoption of a healthy lifestyle in people with MS [39]. Anticipation of positive outcomes, social relationships during physical activity, social activities, and experience of the positive effects of physical activity can improve motivation in people with MS [40]. Besides, a study showed that positive attitude, purposefulness, and planning were factors with positive effects on motivation for engagement in health-related behaviors [49]. In addition to MS-related factors, COVID-19-related factors such as fear, despair, and limited social relationships have reduced motivation for engagement in healthy behaviors.

### Facilitators of HPLBs in people with MS

The two main categories of the facilitators to HPLBs in people with MS were attention to inner abilities and social support. Attention to inner abilities had three subcategories, including self-efficacy, spirituality, and hopefulness. Self-efficacy is a predictor of health in people with MS [50]. It is a psychological mechanism defined as the belief in one’s ability to perform a certain activity even in new, difficult, stressful, or unpredictable conditions. Therefore, self-efficacy can act as a predictor of coping in people with MS [1]. Self-efficacy can affect the process of health promotion through affecting health-related behaviors [51]. A study found that self-efficacy had a significant role in engagement in social activities and management of negative thoughts in people with MS and was a significant predictor of their ability to have independent functioning [1]. Similarly, another study argued that low self-efficacy was a barrier to adherence to physical activity [40]. On the other hand, engagement in self-care activities improves self-efficacy and quality of life and reduces the risks of developing other diseases [39].

Spirituality was another subcategory of attention to inner abilities. Spirituality is like an umbrella that covers all aspects of human life [52]. It is a factor that has positive effects on psychological strains and anxiety; hence, patients with good spiritual health can cope with their conditions more effectively [53]. A study showed that psychological interventions based on spiritual and religious patterns promoted physical and mental health in people with MS [54]. Spiritual health promotion can also improve resilience to COVID-19-related problems and stress [52].

Social support was the second category of the HPLBs facilitators in people with MS. The subcategories of this category were family support, peer support, informational support, healthcare support, and charity support. Social support refers to relationship-based processes that promote health and well-being in stressful conditions and include emotional, informational, and instrumental support [55]. It positively affects coping with diseases and mental health [56]. A main source of social support for people with MS is informal caregivers [57]. Given the significant effects of time limitation caused by competitive demands such as family life and occupation on the adoption of a healthy lifestyle, the family members of people with MS along with other individuals need to provide stronger social support for these people [39]. A study found that despite severe disabilities, most people with MS were highly satisfied with their relationships with their spouses, children, and close friends. Moreover, about 80% of people with MS reported their spouses’ adequate understanding of MS [37]. Another study showed that the spouses of individuals with MS could cope with MS-associated changes and losses and improved their hope and acceptance [58]. In line with previous studies, the participants of the present study placed emphasis on the role of the family, especially the wife, as a source of support. A study showed that social relationships during sport-related activities could improve the motivation for engagement in physical activity [39]. Another study revealed that social support had positive effects on adherence to physical activity in people with MS [41]. Social support improves their coping and sense of belongingness and reduces the effects of MS on their physical and mental health [59].

Informational support was another support-related facilitator of health-related behaviors. A study found that web-based lifestyle education was a good method for providing education to people with MS [60]. Another study showed that online resources were useful in facilitating the patients’ access to rehabilitation services, information, and mental support [61]. Moreover, the results of a study revealed that web-based communication improved the relationship between patients and healthcare providers and enabled people with MS to engage in the process of healthcare-related decision-making more actively [62]. Web-based tele-health methods have received great attention and have played a significant role in facilitating healthcare provision during the COVID-19 pandemic [63] and can be used for remote support and care provision to patients [64]. A study reported the effectiveness of a mobile-based e-learning application in promoting healthy lifestyle in people with MS [64]. These people also wish to have honest and good relationships with their physicians [57]. Such relationships with experts and care providers can significantly improve the motivation of people with MS for health-related behaviors and play a significant role in improving their adherence to a healthy lifestyle [39]. Consistent with the results of previous studies, the participants in the present study were satisfied with the cyberspace where they could ask their questions or share their information about HPLBs. They considered this source of support very necessary, especially during the pandemics.

## Conclusion

This study evaluated the barriers and facilitators of HPLBs in people with MS during the COVID-19 pandemic. The major barriers to HPLBs in these people were lack of knowledge, limited access to resources, and poor health status, and the major facilitators were attention to inner abilities and social support. Therefore, healthcare authorities and policymakers need to consider these findings to develop and apply quality educational and supportive interventions in order to improve knowledge, health literacy, perceived support, self-efficacy, and self-care ability of people living with MS. Such changes may facilitate their engagement in HPLBs and improve their health. This study showed that pandemics could change routine lifestyles and that people living with MS require special attention. Health providers should consider all the physical, psychological and social dimensions of a person in providing their services and design and implement interventions based on comprehensive care.

## Supplementary Information

**Additional file 1:** **Appendix 1.** Consolidated criteria for reporting qualitative studies (COREQ).

## Abbreviations

HPLBs: Health-promoting lifestyle behaviors
MS: Multiple sclerosis
CCA: Conventional content analysis

## Declarations

### Availability of data and materials

The datasets used and analysed during the current study available from the corresponding author on reasonable request.

## References

[1] Dymecka J, Gerymski R, Tataruch R, Bidzan M (2021). Fatigue, physical disability and self-efficacy as predictors of the acceptance of illness and health-related quality of life in patients with multiple sclerosis. Int J Enviro Res Public Health.

[2] Dobson R, Giovannoni G (2019). Multiple sclerosis–a review. Eur J Neurol.

[3] Amini P, Almasi-Hashiani A, Sahraian MA, Najafi M, Eskandarieh S (2021). Multiple sclerosis projection in Tehran, Iran using Bayesian structural time series. BMC Neurol.

[4] Hauser SL, Cree BA (2020). Treatment of multiple sclerosis: a review. Am J Med.

[5] Giovannetti AM, Brambilla L, Torri Clerici V, Antozzi C, Mantegazza R, Černiauskaitė M (2017). Difficulties in adjustment to multiple sclerosis: vulnerability and unpredictability of illness in the foreground. Disabil Rehabil.

[6] Strober LB, Becker A, Randolph JJ (2018). Role of positive lifestyle activities on mood, cognition, well-being, and disease characteristics in multiple sclerosis. Appl Neuropsychol Adult.

[7] Penwell-Waines L, Lewis K, Valvano A, Smith S, Rahn R, Stepleman L (2017). Testing the health promotion model for adherence and quality of life in individuals with multiple sclerosis. Psychol Health Med.

[8] Alizadeh T, Keshavarz Z, Mirghafourvand M, Zayeri F (2018). Investigation of health promoting lifestyle and social support and their correlation among Iranian women with multiple sclerosis. Int J Women’s Health Reprod Sci.

[9] Jafari Jovzani R, Mosavi SAM, Ahmadi A, Asgari N (2016). Comparing Executive Function and Life orientation in Multiple Sclerosis Patients and Healthy People. J Sabzevar Univ Med Sci.

[10] Hanna M, Strober LB (2020). Anxiety and depression in Multiple Sclerosis (MS): Antecedents, consequences, and differential impact on well-being and quality of life. Mult Scler Relat Disord.

[11] Valery PC, Ibiebele T, Harris M, Green AC, Cotterill A, Moloney A, et al. Diet, physical activity, and obesity in school-aged Indigenous youths in northern Australia. J Obes. 2012;2012:893508. 10.1155/2012/893508 PMC3376785 22720140

[12] Mahmoudi H, Asghari Jafarabadi M, Babazadeh T, Shirzadi S, Mohammadi Y, Sharifi Saqezi P (2015). Health promoting behaviors in pregnant women admitted to the prenatal care unit of Imam Khomeini hospital of Saqqez. J Educ Community Health.

[13] Hosseini M, Ashktorab T, Taghdisi MH, Khodayari MT (2017). The interpersonal influences as a factor for health promoting life style in nursing students: a mixed method study. Glob J Health Sci.

[14] Nandi A, Glymour MM, Subramanian S (2014). Association among socioeconomic status, health behaviors, and all-cause mortality in the United States. Epidemiology.

[15] Fitzgerald KC, Tyry T, Salter A, Cofield SS, Cutter G, Fox R (2018). Diet quality is associated with disability and symptom severity in multiple sclerosis. Neurology.

[16] Russell RD, Black LJ, Begley A (2021). Navigating dietary advice for multiple sclerosis. Health Expect.

[17] Motl RW (2020). Exercise and multiple sclerosis. Phys Exerc Hum Health.

[18] Asadi HM, Zahrakar K,  Farzad V (2019). Effectiveness of Cognitive-Behavioral Stress Management Training (CBSM) on reducing stress symptoms of women with Multiple Sclerosis (MS).

[19] Bonavita S, Sparaco M, Russo A, Borriello G, Lavorgna L (2021). Perceived stress and social support in a large population of people with multiple sclerosis recruited online through the COVID-19 pandemic. Eur J Neurol.

[20] Khajehnasiri F, Hokmabadi E, Lotfi M, Dabiran S, Khosravi S, Sharifi N (2022). Study of health-promoting lifestyle and its effective factors in the employees of tobacco company. Iran J Health Educ Health Prom.

[21] Mascherini G, Catelan D, Pellegrini-Giampietro DE, Petri C, Scaletti C, Gulisano M (2021). Changes in physical activity levels, eating habits and psychological well-being during the Italian COVID-19 pandemic lockdown: Impact of socio-demographic factors on the Florentine academic population. PLoS ONE.

[22] Mares J, Hartung HP (2020). Multiple sclerosis and COVID-19. Biomed Pap Med Fac Univ Palacky, Olomouc Czech.

[23] Alotaibi N, Al-Sayegh N, Nadar M, Shayea A, Allafi A, Almari M (2021). Investigation of health science students’ knowledge regarding healthy lifestyle promotion during the spread of COVID-19 pandemic: a randomized controlled trial. Front Public Health.

[24] [Available from: https://covid19.who.int/.

[25] Roberts NJ, McAloney-Kocaman K, Lippiett K, Ray E, Welch L, Kelly C (2021). Levels of resilience, anxiety and depression in nurses working in respiratory clinical areas during the COVID pandemic. Respir Med.

[26] Saadat S, Kalantari M, Kajbaf MB, Hosseininezhad M (2019). A comparative study of health promoting behaviors in healthy individuals and patients with multiple sclerosis: an analytical study. Journal of Hayat.

[27] Sikes EM, Richardson EV, Motl RW (2019). A qualitative study of exercise and physical activity in adolescents with pediatric-onset multiple sclerosis. Int J MS Care.

[28] Pilutti L, Dlugonski D, Sandroff B, Klaren R, Motl R (2014). Randomized controlled trial of a behavioral intervention targeting symptoms and physical activity in multiple sclerosis. Mult Scler J.

[29] Riemann-Lorenz K, Motl RW, Casey B, Coote S, Daubmann A, Heesen C (2021). Possible determinants of long-term adherence to physical activity in multiple sclerosis-theory-based development of a comprehensive questionnaire and results from a German survey study. Disabil Rehabil.

[30] Plow M, Finlayson M, Cho C (2012). Correlates of nutritional behavior in individuals with multiple sclerosis. Disabil Health J.

[31] Zhang W, Becker H, Stuifbergen AK, Brown A (2017). Predicting health promotion and quality of life with symptom clusters and social supports among older adults with multiple sclerosis. J Gerontol Nurs.

[32] Tol A, Esmaeili Shahmirzadi S, Shojaeizadeh D, Eshraghian MR, Mohebbi B (2012). Determination Of perceived barriers and benefits of adopting health-promoting behaviors in cardiovascular diseases prevention: application of preventative behavior model. Payavard Salamat.

[33] Hsieh H-F, Shannon SE (2005). Three approaches to qualitative content analysis. Qual Health Res.

[34] de Rezende LFM, Rey-López JP, Matsudo VKR, do Carmo Luiz O (2014). Sedentary behavior and health outcomes among older adults: a systematic review. BMC public health..

[35] Grove SK, Gray JR, Sutherland S (2017). Burns and Grove's the practice of nursing research: appraisal, synthesis, and generation of evidence: Elsevier.

[36] Graneheim UH, Lundman B (2004). Qualitative content analysis in nursing research: concepts, procedures and measures to achieve trustworthiness. Nurse Educ Today.

[37] Herbert LB, Zerkowski K, O’Brien S, Leonard KV, Bhowmick A (2019). Impact on interpersonal relationships among patients with multiple sclerosis and their partners. Neurodegener Dis Manag.

[38] Ploughman M (2017). Breaking down the barriers to physical activity among people with multiple sclerosis–a narrative review. Phys Ther Rev.

[39] Barnard E, Brown CR, Weiland TJ, Jelinek GA, Marck CH (2020). Understanding barriers, enablers, and long-term adherence to a health behavior intervention in people with multiple sclerosis. Disabil Rehabil.

[40] Riemann-Lorenz K, Wienert J, Streber R, Motl RW, Coote S, Heesen C (2020). Long-term physical activity in people with multiple sclerosis: exploring expert views on facilitators and barriers. Disabil Rehabil.

[41] Fifolt M, Richardson EV, Barstow E, Motl RW (2020). Exercise behaviors of persons with multiple sclerosis through the stepwise implementation lens of social cognitive theory. Disabil Rehabil.

[42] Messmer Uccelli M, Traversa S, Trojano M, Viterbo RG, Ghezzi A, Signori A (2013). Lack of information about multiple sclerosis in children can impact parents' sense of competency and satisfaction within the couple. J Neurol Sci.

[43] Zahraie S, Amini S, Saebi S (2018). The relationship between illness perception, stigma and cognitive fusion with quality of life of the women with multiple sclerosis. J Psychol Stud.

[44] Zhang GX, Sanabria C, Martínez D, Zhang WT, Gao SS, Alemán A (2021). Social and professional consequences of COVID-19 lockdown in patients with multiple sclerosis from two very different populations. Neurologia (Barcelona, Spain).

[45] Danhieux K, Buffel V, Pairon A, Benkheil A, Remmen R, Wouters E (2020). The impact of COVID-19 on chronic care according to providers: a qualitative study among primary care practices in Belgium. BMC Fam Pract.

[46] Khajeheian D, Labafi S, Omidi A (2019). Media and health: identifying and analyzing news frames in media representation of MS disease. J Cult Commun Stud.

[47] Amani F, Hoseinzadeh S, Sabzvari A, Avesta L, Kahnamouei-Aghdam F, Barak M (2015). Awareness rate of Ardabil city people about multiple sclerosis. Int J Adv Med.

[48] Khoshravesh S, Taymoori P, Abdolmaleki S, Khomand P, Pavara M (2016). Awareness, risk perception, and protective behaviors in regard to multiple sclerosis among people in Sanandaj Iran. Sci J Kurdistan Univ Med Sci.

[49] Plow MA, Golding M (2016). A qualitative study of multiple health behaviors in adults with multiple sclerosis. Int J MS Care.

[50] Shaker Devlagh A, Aminpoor M (2015). Optimism-pessimism and self-efficacy in the patients with multiple sclerosis. J Health Care.

[51] Gameian D, Hosseinsabet F, Motamedi A (2018). Effectiveness of brief solution-focused group therapy on quality of life and self-efficacy of multiple sclerosis women. J Clin Psychol.

[52] Taghdisi MH, Azarbayejani M, Khayamzadeh M, Lankarani KB (2021). The importance of spiritual health in the COVID-19. J Cult Health Prom.

[53] Madmoli Y, Ahmadi Y, Shamloo MBB, Rostami F, Khodadadi M, Raji M (2018). Spiritual health and its related factors in patients with thalassemia major. Med Ethics J.

[54] Abdekhodaie Z, Shahidi S, Mazaheri MA, Panaghi L, Nejati V (2018). Spiritual/religious pattern in patient with multiple sclerosis: a qualitative study. J Psychol.

[55] Lloyd-Jones B (2021). Developing competencies for emotional, instrumental, and informational student support during the COVID-19 pandemic: a human relations/human resource development approach. Adv Dev Hum Resour.

[56] Rommer PS, Sühnel A, König N, Zettl UK (2017). Coping with multiple sclerosis-the role of social support. Acta Neurol Scand.

[57] Strupp J, Voltz R, Golla H (2016). Opening locked doors: Integrating a palliative care approach into the management of patients with severe multiple sclerosis. Multiple sclerosis (Houndmills, Basingstoke, England).

[58] Appleton D, Robertson N, Mitchell L, Lesley R (2018). Our disease: a qualitative meta-synthesis of the experiences of spousal/partner caregivers of people with multiple sclerosis. Scand J Caring Sci.

[59] Jadid Milani M, Ashktorab T, Abed Saaidi Z, Alavi Majd H (2013). Promotion of illness perception and it’s aspects in a Multiple Sclerosis (MS) peer support groups. Knowledge Health.

[60] Bevens W, Weiland TJ, Gray K, Neate SL, Nag N, Simpson-Yap S (2022). The feasibility of a web-based educational lifestyle program for people with multiple sclerosis: a randomized controlled trial. Front Public Health.

[61] Farpour HR, Hoveidaei AH, Habibi L, Moosavi M, Farpour S (2020). The impact of social media use on depression in multiple sclerosis patients. Acta Neurol Belg.

[62] Kantor D, Bright JR, Burtchell J (2018). Perspectives from the patient and the healthcare professional in multiple sclerosis: social media and participatory medicine. Neurology and therapy.

[63] AbdulRahman M, Al-Tahri F, AlMehairi MK, Carrick FR, Aldallal AMR (2022). Digital health technology for remote care in primary care during the COVID-19 pandemic: experience from Dubai. Telemed e-Health.

[64] d’Arma A, Rossi V, Pugnetti L, Grosso C, Sinatra M, Dos Santos R (2021). Managing chronic disease in the COVID-19 pandemic: an e-learning application to promote a healthy lifestyle for persons with multiple sclerosis. Psychol Health Med.

